# Mpox Epidemiology and Risk Factors, Nigeria, 2022

**DOI:** 10.3201/eid3009.240135

**Published:** 2024-09

**Authors:** Dimie Ogoina, Mahmmod Muazu Dalhat, Ballah Akawu Denue, Mildred Okowa, Nneka Marian Chika-Igwenyi, Sebastine Oseghae Oiwoh, Ekaete Alice Tobin, Hakeem Abiola Yusuff, Anastacia Okwudili Ojimba, Umenzekwe Chukwudi Christian, John-Tunde Aremu, Simji Samuel Gomerep, Kambai Lalus Habila, Sati Klein Awang, Olukemi Adekanmbi, Michael Iroezindu, Asukwo Onukak, Olanrewaju Falodun, Mogaji Sunday, Simon Mafuka Johnson, Abimbola Olaitan, Chizaram Onyeaghala, Datonye Alasia, Juliet Mmerem, Uche Unigwe, Vivian Kwaghe, Mukhtar Abdulmajid Adeiza, on behalf of Nigerian Infectious Diseases Society (NIDS) mpox study group

**Affiliations:** Niger Delta University/Niger Delta University Teaching Hospital, Bayelsa, Nigeria (D. Ogoina);; Infectious Diseases Control Centre, Kaduna State, Nigeria (M.M. Dalhat);; University of Maiduguri, Maiduguri, Nigeria (B.A. Denue); Ministry of Health, Asaba, Nigeria (M. Okowa);; Alex Ekwueme Federal University Teaching Hospital, Abakaliki, Nigeria (N.M. Chika-Igwenyi);; Irrua Specialist Teaching Hospital, Irrua, Nigeria (S. Oiwoh, E.A. Tobin);; Ministry of Health, Abeokuta, Nigeria (H.A. Yusuff);; Federal Medical Centre, Asaba (A.O. Ojimba);; Nnamdi Azikiwe University Teaching Hospital, Nnewi, Nigeria. (U.C. Christian);; Federal Teaching Hospital, Gombe, Nigeria (J.T. Aremu);; Jos University Teaching hospital/University of Jos, Jos, Nigeria (S.S. Gomerep);; Kaduna State Emergency Medical Services and Ambulance System, Kaduna, Nigeria (K.L. Habila);; Modibo Adamawa University Teaching Hospital, Yola, Nigeria (S.K. Awang);; University of Ibadan College of Medicine, Ibadan, Nigeria (O. Adekanmbi);; University of Nigeria Teaching Hospital, Enugu, Nigeria (M. Iroezindu, J Mmerem, U. Unigwe);; University of Uyo, Uyo, Nigeria (A. Onukak);; National Hospital Abuja, Abuja, Nigeria (O. Falodun);; Federal Medical Centre Ebute-Metta, Lagos, Nigeria (M. Sunday);; Federal University Teaching Hospital, Imo, Nigeria (S.M. Johnson);; Olabisi Onabanjo University Teaching Hospital, Sagamu, Nigeria (A. Olaitan);; University of Port Harcourt Teaching Hospital, Port Harcourt, Nigeria (C. Onyeaghala, D. Alasia);; University of Abuja Teaching Hospital, Gwagalada, Nigeria (V. Kwaghe);; Ahmadu Bello University Teaching Hospital, Zaria, Nigeria (M.A. Adeiza)

**Keywords:** mpox, monkeypox virus, varicella zoster virus, risk factors, epidemiology, predictors, sexual transmission, animal exposure, viruses, Nigeria, zoonoses

## Abstract

To investigate epidemiology of and risk factors for laboratory-confirmed mpox during the 2022 outbreak in Nigeria, we enrolled 265 persons with suspected mpox. A total of 163 (61.5%) were confirmed to have mpox; 137 (84.0%) were adults, 112 (68.7%) male, 143 (87.7%) urban/semi-urban dwellers, 12 (7.4%) self-reported gay men, and 3 (1.8%) female sex workers. Significant risk factors for adults were sexual and nonsexual contact with persons who had mpox, as well as risky sexual behavior. For children, risk factors were close contact with an mpox-positive person and prior animal exposure. Odds of being mpox positive were higher for adults with HIV and lower for those co-infected with varicella zoster virus (VZV). No children were HIV-seropositive; odds of being mpox positive were higher for children with VZV infection. Our findings indicate mpox affects primarily adults in Nigeria, partially driven by sexual activity; childhood cases were driven by close contact, animal exposure, and VZV co-infection.

Human mpox is a zoonotic disease caused by 2 distinct clades (I and II) of the monkeypox virus (MPXV) (*1*). Clade I primarily affects children and adolescents in Central Africa, especially in the Democratic Republic of Congo (DRC) (*1*,*2*). Clade IIa was responsible for the 2003 human outbreak of mpox in the United States, and clade IIb caused the 2017–2019 mpox outbreak in Nigeria and the 2022 global outbreak (*1*); ≈92,000 confirmed cases and 171 deaths were reported in 116 countries as of December 22, 2023 (*3*).

The epidemiologic characteristics of mpox during the 2022 outbreak have been described (*4*–*8*). The evidence suggests that ≈96% of mpox cases during the 2022 outbreak were in men, mostly 20­­­–41 years of age, and the predominant mode of transmission (≈80%) was sexual encounter. Furthermore, the most frequent independent predictors of laboratory-confirmed mpox during the 2022 mpox outbreak have been identified as being male; being gay, bisexual, and other men who have sex with men (GBMSM); being a person living with HIV (PLHIV); having multiple sex partners; and having lesions in the anogenital area (*9*–*14*).

The 2017–2019 mpox outbreak in Nigeria predominantly affected young urban adults; human-to-human and zoonotic-related transmissions were suspected (*15*). Confirmed cases were reported among prison inmates, household and sexual contacts, and persons exposed to wildlife (*15*). The 2017–2019 outbreak provided the first documented evidence of mpox transmission via sexual contact and of mpox being associated with having multiple sex partners or advanced HIV disease (*16*–*18*). However, in ≈60% of cases, the risk factors or sources of exposure for mpox were unknown, suggesting a substantial knowledge gap in the epidemiology of mpox in Nigeria (*15*).

During the 2022 mpox outbreak, ≈1,400 cases were reported in Africa, of which Nigeria accounted for 42% (*3*). However, only a few studies from Africa discuss the epidemiology of and risk factors for laboratory-confirmed mpox infections during that outbreak. A case series from Nigeria described the interplay of mpox with varicella zoster virus (VZV) but was limited to southern Nigeria (*19*), suggesting the need to explore the co-infection on a national scale. Since 2023, the DRC has reported increasing mpox cases, including those caused by the sexually transmitted clade I strain (*20*,*21*). That change in the epidemiology is concerning and calls for concerted action and more information about the epidemiology of and risk factors for mpox in countries in Africa where the disease was previously endemic.

To investigate the epidemiology of and risk factors for laboratory-confirmed mpox in Nigeria during the 2022 outbreak, we conducted an observational cross-sectional study to address existing knowledge gaps and provide insights that can be used to develop public health strategies and interventions to control future mpox outbreaks.

## Methods

### Ethics Statement

We obtained ethics approval for the study from the National Health Research Ethics Committee, Nigeria (NHREC/01/01/2007–25/10/2022). All participants gave informed consent to participate in the study.

### Study Participants

Our cross-sectional study included persons with suspected mpox who attended mpox treatment centers and outpatient clinics across Nigeria during June 1–December 30, 2022. We defined a suspected case of mpox by using the Nigeria Centre for Disease Control and Prevention guidelines, as previously described (*22*). On the basis of an average of 12 suspected cases of mpox seen monthly during January–April 2022 in Nigeria, we estimated a minimum sample size of 158 participants, including a 10% dropout rate. We invited all mpox treatment centers and outpatient clinics across Nigeria to participate in the study and consecutively enrolled persons with suspected mpox who attended study sites and gave informed verbal or written consent. Suspected mpox was diagnosed by PCR at the National Reference Laboratory of the Nigeria Centre for Disease Control and Prevention as previously described (*23*). We defined an mpox-positive participant as an mpox-suspected participant for whom MPXV infection was confirmed by real-time PCR. Because of lack of laboratory diagnoses, we excluded probable cases of mpox (Appendix).

To document epidemiologic and clinical variables of all study participants, we used a structured case report form, which was developed from existing mpox literature review (*4*,*15*,*18*,*23*–*25*) and included variables such as patient age, sex, occupation, sexual orientation, and potential routes and risk factors for mpox transmission (e.g., animal exposure, close contact, and sexual behavior). Sexual history was not obtained for all children. We also documented comorbidities (e.g., HIV and VZV co-infections) (Appendix). All variables were documented at manifestation or at the time of participant recruitment.

We analyzed study data by using the SPSS Statistics 26 (IBM, https://www.ibm.com). We summarized categorical variables as frequencies and percentages and summarized continuous variables by using median and interquartile ranges (IQRs) because of nonnormal distribution. We used χ^2^ for categorical variables (or Fisher exact tests when assumptions for χ^2^ were not met because of small sample size) and Mann-Whitney tests (comparing median values) to determine variables associated with being mpox positive. We determined independent predictors of mpox positivity separately by using logistic regression models that included significant epidemiologic variables on univariate analysis and other relevant variables known to be theoretically associated with mpox infection from prior literature. We deleted missing variables pairwise without replacements. We excluded educational level from the model because of strong correlation with age group. Because of missing data, we did not include HIV and VZV data in the logistic models. The logistic regression tables detail the variables included in each model. In view of differences in epidemiologic characteristics across age groups, we assessed the risk factors for mpox positivity for the entire study population and separately for children (<18 years of age) and adults. We reported results as crude odd ratios (ORs) and adjusted odds ratios (aORs) with 95% CIs. We considered p<0.05 (2-tailed) as statistically significant.

## Results

### Study Population

We enrolled 280 persons with suspected cases of mpox during the study period, among whom we excluded 15 (5.4%) from the final analysis because of missing data related to sociodemographic and epidemiologic characteristics. We enrolled 265 study participants, 28 days–69 years of age (median 27 years, IQR 14–36 years) across 23 states and the Federal Capital Territory in Nigeria (Figure). Of the 265 participants, 163 (61.5%) were mpox positive and 102 (38.5%) were mpox negative (Table 1). The mpox-positive participants (median age 30 years [IQR 22–37 years]) were older than the mpox-negative participants (median age 19 years [IQR 8–32 years]; p<0.0001).

**Figure:**
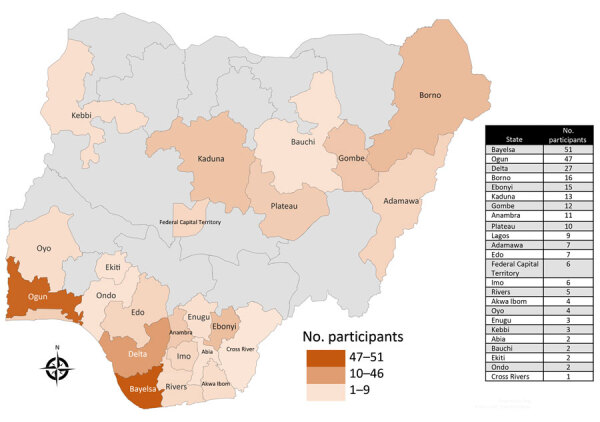
Geographic distribution of sites in Nigeria participating in a study of epidemiology and risk factors for laboratory-confirmed mpox during the mpox outbreak, Nigeria, 2022. A total of 265 study participants were enrolled from all geopolitical zones of the country, across 23 states and the Federal Capital Territory in Nigeria.

**Table 1:** Demographic characteristics and epidemiologic risk factors for PCR-confirmed mpox among adults and children during mpox outbreak, Nigeria, 2022*

Study variable	No. (%) patients				Univariate analysis			Multivariate analysis	
	Total	Mpox-pos	Mpox-neg		cOR (95% CI)	p value†		aOR (95% CI)	p value†
Age group									**<0.0001**
Child, <18 y	73 (27.5)	26 (16.0)	47 (46.1)		Referent			Referent	
Young adult, 18–35 y	125 (47.2)	88 (54.0)	37 (36.3)		4.30 (2.33–7.94)	**<0.0001**		3.93 (2.06–7.50)	**<0.0000**
Older adult, >35 y	67 (25.3)	49 (30.0)	18 (17.6)		4.92 (2.39–10.13)	**<0.0001**		4.75 (2.23–10.13)	**<0.0001**
Sex at birth									
M	174 (65.7)	112 (68.7)	62 (60.8)		1.42 (0.84–2.38)	0.186		1.38 (0.77–2.48)	0.284
F	91 (34.3)	51 (31.3)	40 (39.2)		Referent			Referent	
Local travel									
Yes	34 (12.8)	28 (17.2)	6 (5.9)		3.32 (1.32–8.33)	**0.007**		2.02 (0.76–5.34)	0.158
No	231 (87.2)	135 (82.8)	96 (94.1)		Referent			Referent	
Close contact with person with confirmed mpox									
Yes	46 (17.4)	36 (22.1)	10 (9.8)		2.61 (1.21–5.52)	**0.010**		2.96 (1.26–6.96)	**0.013**
No	219 (82.6)	127 (77.9)	92 (90.2)		Referent			Referent	
Prior animal exposure									
Yes	43 (16.2)	35 (21.5)	8 (7.8)		3.21 (1.30–7.24)	**0.003**		2.35 (0.97–5.66)	0.058
No	222 (83.8)	128 (78.5)	94 (92.2)		Referent			Referent	
Residence									
Urban/semi-urban	231 (87.2)	143 (87.7)	88 (86.3)		1.14 (0.55–2.37)	0.73			
Rural	34 (12.8)	20 (12.3)	14 (13.7)		Referent				
Married‡									
Ever	95 (49.5)	70 (51.1)	25 (45.5)		1.25 (0.67–2.35)	0.48			
Never	97 (50.5)	67 (48.9)	30 (54.5)		Referent				
Education									
None	59 (22.3)	35 (21.5)	24 (41.4)		Referent				
Primary	32 (12.1)	12 (7.4)	20 (34.5)		0.41 (0.17–0.99)	**0.003**			
Secondary	68 (25.7)	39 (23.9)	29 (50)		0.92 (0.45–1.87)				
Tertiary	106 (40.0)	77 (47.2)	29 (50)		1.82 (0.93–3.57)				
International travel									
Yes	3 (1.1)	1 (0.6)	2 (2)		0.31 (0.03–3.45)	0.561§			
No	262 (98.9)	162 (99.4)	100 (98)		Referent				
Close contact with person with suspected mpox									
Yes	78 (29.4)	59 (36.2)	19 (18.6)		2.48 (1.37–4.48)	**0.002**			
No	187 (70.6)	104 (63.8)	83 (81.4)		Referent				
Care of person with suspected mpox									
Yes	5 (1.9)	4 (2.5)	1 (1)		2.54 (0.28–23.06)	0.652§			
No	260 (98.1)	159 (97.5)	101 (99)		Referent				
Prior smallpox vaccine									
Yes	13 (4.9)	9 (5.5)	4 (3.9)		1.43 (0.43–4.79)	0.557			
No	252 (95.1)	154 (94.5)	98 (96.1)		Referent				
Past chickenpox¶									
Yes	23 (16.6)	16 (17.4)	7 (12.7)		1.44 (0.55–3.77)	0.451			
No	124 (84.4)	76 (82.6)	48 (87.3)		Referent				
HIV-status¶									
Positive	26 (12.2)	24 (18.0)	2 (2.5)		8.59 (1.97–37.4)	**0.001**			
Negative	187 (87.8)	109 (82.0)	78 (97.5)		Referent				
VZV status¶									
Positive	86 (36.4)	55 (35.9)	31 (37.3)		0.94 (0.54–1.64)	0.831			
Negative	150 (63.6)	98 (64.1)	52 (62.7)		Referent				
Other comorbidities#									
Yes	13 (4.9)	10 (6.1)	3 (2.9)		2.16 (0.58–8.03)	0.241			
No	252 (95.1)	153 (93.9)	99 (97.1)		Referent				

*Boldface indicates statistical significance. aOR, adjusted odds ratio; cOR, crude odd ratio; neg, negative; pos, positive; VZV, varicella zoster virus. †p values determined by χ^2^ tests except as indicated. ‡Marital status data included adults only. §Fisher exact test. ¶Some past chickenpox, VZV, and HIV status data were missing. #Other comorbidities include type 2 diabetes mellitus, hypertension, and sickle cell disease.

### Demographic and Epidemiologic Characteristics of Mpox-Positive Participants

Of the 163 mpox-positive participants, 137 (84.0%) were adults, 112 (68.7%) were male, 143 (87.7%) were urban/semi-urban dwellers, 12 (7.4%) were self-reported GBMSM, and 3 (1.8%) were female sex workers (Appendix Table). Among the 163 mpox-positive participants, exposure was unknown for 87 (53.4%) and >1 exposure was reported for 76 (46.6%). Specifically, 59 (36.2%) had contact with a person with a suspected case, 36 (22.1%) had close contact with a person with a confirmed case, 35 (21.5%) reported animal exposure, and 35 (21.5%) had sexual contact with a person with a suspected case. Of the 46 mpox-positive participants who provided information about possible places of exposure, 23 (50%) were exposed at home, 20 (43.5%) in the community, and 3 (6.5%) in the hospital. Among the mpox-positive participants tested for HIV and VZV co-infections, 35.9% (55/153) had VZV co-infection, 18.0% (24/133) had HIV co-infection, and 6.0% (8/133) had both HIV and VZV co-infections. Among the 102 mpox-negative participants, 31 (30.4%) were VZV positive, 52 (50.9%) were VZV negative, and 19 (18.6%) were missing data on VZV status. We did not investigate the causes of skin rash among participants who were VZV-negative and those for whom VZV status was missing.

Among the 23 study participants who reported a history of chickenpox (Table 1), 7 (30.4%) were VZV positive; 2 were mpox-negative adults and 5 were mpox positive (a 16-year-old adolescent and 4 adults), all of whom were probably experiencing reactivated herpes zoster infection. Among the 2 mpox-negative adults with probable reactivated herpes zoster infection was a recently diagnosed 63-year-old man living with HIV whose CD4 cell count was unknown/missing.

### Epidemiologic Risk Factors for Mpox Positivity

Univariate analysis indicated that the epidemiologic risk factors associated with mpox positivity among the 265 study participants were history of prior animal exposure, age group, close contact with a person with confirmed mpox, educational level, and local travel (Table 1). In a logistic regression model that included age group, sex, animal exposure, close contact with confirmed case and local travel, the independent predictors of mpox positivity were age group and close contact with a person with confirmed mpox (Table 1). The odds of being mpox positive were significantly higher among younger adults (18–35 years of age) (aOR 3.93, 95% CI 2.06–7.50) and older adults (>35 years of age) (aOR 4.75, 95% CI 2.23–10.13) than among children. Odds of being mpox positive were significantly higher among participants who had reported close contact with a person with confirmed mpox than among those who had not (aOR 2.96, 95% CI 1.26–6.96). Among 213 participants with known HIV status, odds of being mpox positive were greater among PLHIV than among those who were HIV negative (OR 8.59, 95% CI 1.97–37.40; p = 0.001).

### Epidemiologic Risk Factors among Adults

Univariate analysis indicated that among the 192 adult participants, the variables associated with being mpox positive were prior sexual and nonsexual contact with a person with suspected mpox and recent history of risky sexual behavior (Table 2). The independent predictors among adults for being mpox positive included recent history of risky sexual behavior (aOR 2.81, 95% CI 1.40–5.63), nonsexual contact with a person with a suspected case (aOR 5.50, 95% CI 1.12–27.14), and sexual contact with a person with a suspected case (aOR 2.81, 95% CI 1.01–7.79) (Table 2). Among the 142 adults with known HIV status and the 172 with known VZV status, the odds of being mpox positive were significantly higher among PLHIV (OR 4.77, 95% CI 1.07–21.34) and significantly lower among those who were VZV positive (OR 0.43, 95% CI 0.21–0.87). History of having had multiple sex partners, having had sex recently, and having engaged in risky sexual behaviors were significantly associated with being mpox positive (Table 3).

**Table 2:** Demographic characteristics and epidemiologic risk factors for PCR-confirmed mpox among adults during the mpox outbreak, Nigeria, 2022*

Variables	No. (%) patients				Univariate analysis			Multivariate analysis	
	Total	Mpox-pos	Mpox-neg		cOR (95% CI)	p value†		aOR (95% CI)	p value†
Age group,									
Young adult, 18–35 y	125 (65.1)	88 (64.2)	37 (67.3)		0.87 (0.45–1.70)	0.69		1.36 (0.66– 2.80)	0.409
Older adult, >35 y	67 (34.9)	49 (35.8)	18 (32.7)		Referent			Referent	
Sex at birth									
M	131 (68.2)	95 (69.3)	36 (65.5)		1.19 (0.61–2.32)	0.601		1.29 (0.59– 2.79)	0.523
F	61 (31.8)	42 (30.7)	19 (34.5)		Referent			Referent	
Nonsexual contact with person with suspected mpox									
Yes	17 (8.90)	15 (10.9)	2 (3.6)		3.26 (0.72–14.75)	0.158		5.50 (1.12–27.14)	**0.036**
No	175 (91.1)	122 (89.1)	53 (96.4)		Referent			Referent	
Sexual contact with person with suspected mpox									
Yes	41 (21.4)	35 (25.5)	6 (10.9)		2.80 (1.10–7.11)	**0.025**		2.81 (1.01–7.79)	**0.048**
No	151 (78.6)	102 (74.5)	49 (89.1)		Referent			Referent	
Risky sexual behavior‡									
Yes	113 (58.9)	90 (65.7)	23 (41.8)		2.66 (1.40–5.06)	**0.002**		2.808 (1.40–5.3)	**0.004**
No	79 (41.1)	47 (34.3)	32 (58.2)		Referent			Referent	
Animal exposure									
Yes	36 (18.8)	30 (21.9)	6 (10.9)		2.29 (0.89–5.86)	0.078		1.79 (0.66–4.88)	0.255
No	156 (81.2)	107 (78.1)	49 (89.1)		Referent			Referent	
Local travel									
Yes	33 (17.2)	27 (19.7)	6 (10.9)		2.00 (0.78–5.17)	0.144		1.74 (0.64–4.72)	0.28
No	159 (82.8)	110 (80.3)	49 (89.1)		Referent			Referent	
Residence									
Urban/semi-urban	170 (88.5)	122 (89.1)	48 (87.3)		1.19 (0.46–3.09)	0.727			
Rural	22 (11.5)	15 (10.9)	7 (12.7)		Referent				
Married									
Ever	95 (49.5)	70 (51.1)	25 (45.5)		1.25 (0.67–2.35)	0.48			
Never	97 (50.5)	67 (48.9)	30 (54.5)		Referent				
Education									
None	25 (13.0)	21 (15.3)	4 (7.3)		Referent				
Primary	8 (4.20)	5 (3.60)	3 (5.5)		0.32 (0.05–1.90)	0.21			
Secondary	54 (28.1)	34 (24.8)	20 (36.4)		0.32 (0.10–1.08)	0.07			
Tertiary	105 (54.7)	77 (56.2)	28 (50.9)		0.53 (0.17–1.66)	0.27			
International travel									
Yes	3 (1.60)	1 (0.70)	2 (3.6)		0.19 (0.02–2.19)	0.198§			
No	189 (98.4)	136 (99.3)	53 (96.4)		Referent				
Close contact with confirmed mpox									
Yes	33 (17.2)	29 (21.2)	4 (7.3)		3.42 (1.14–10.26)	**0.021**			
No	159 (82.8)	108 (78.8)	51 (92.7)		Referent				
GBMSM									
No	146 (76.0)	107 (78.1)	39 (70.9)		Referent				
Yes	15 (7.80)	12 (8.80)	3 (5.5)		1.46 (0.39–5.44)	0.575			
Unknown	31 (16.1)	18 (13.1)	13 (23.6)		0.51 (0.23–1.13)	0.095			
Care of person with suspected mpox									
Yes	5 (2.60)	4 (2.90)	1 (1.8)		1.02 (0.18–14.86)	0.999§			
No	187 (97.4)	133 (97.1)	54 (98.2)		Referent				
Prior smallpox vaccine									
Yes	13 (6.80)	9 (6.60)	4 (7.3)		0.90 (0.26–3.04)	0.999§			
No	179 (93.2)	128 (93.4)	51 (92.7)		Referent				
Comorbidities¶									
Yes	13 (6.80)	10 (7.30)	3 (5.5)		0.87 (0.23–3.30)	0.761§			
No	179 (93.2)	127 (92.7)	52 (94.5)		Referent				
HIV status#									
Positive	26 (18.3)	24 (22.4)	2 (5.7)		4.71 (1.07–21.34)	**0.026**			
Negative	116 (81.7)	83 (77.6)	33 (94.3)		Referent				
VZV status									
Positive	62 (36.0)	40 (31.0)	22 (51.2)		0.43 (0.21–0.87)	**0.017**			
Negative	110 (64.0)	89 (69.0)	21 (48.8)		Referent				

*Boldface indicates statistical significance, Empty cells indicate not applicable. aOR, adjusted odds ratio; cOR, crude odds ratio; GBMSM, gay and bisexual and men who have sex with men; neg, negative; pos, positive; VZV, varicella zoster virus. †p values determined by χ^2^ tests except as indicated. ‡Risky sexual behavior is defined as >1 of the following in the 3 mo before illness onset: condomless casual sex; multiple sexual partners (>2 more concurrent sex partners); treatment for sexually transmitted infections; transactional sex (payment for sex); sex with a sex worker. §Fisher exact test. ¶Comorbidities include type 2 diabetes mellitus, hypertension, and sickle cell disease. #Some HIV and VZV status data were missing.

**Table 3:** Univariate analysis of associated between sexual histories of adults and mpox-PCR status during mpox outbreak, Nigeria, 2022*

Variables†	No. (%)			cOR (95% CI)	p value‡
	Mpox positive	Mpox negative	Total		
Condomless casual sex, n = 166					
Yes	77 (62.1)	19 (45.2)	96 (57.8)	1.98 (0.98–4.02)	0.056
No	47 (37.9)	23 (54.8)	70 (42.2)		
Multiple sexual partners, n = 170					
Yes	65 (50.8)	10 (23.8)	75 (44.1)	3.30 (1.50–7.28)	**0.002**
No	63 (49.2)	32 (76.2)	95 (55.9)		
Sex with sex worker, n = 166					
Yes	23 (18.5)	4 (9.5)	27 (16.3)	2.16 (0.70–6.67)	0.228
No	101 (81.5)	38 (90.5)	139 (83.7)		
Transactional sex, n = 103					
Yes	6 (8.10)	2 (6.9)	8 (7.80)	1.19 (0.23–6.27)	0.99
No	68 (91.9)	27 (93.1)	95 (92.2)		
Sex in prior month, n = 192					
Yes	101 (73.7)	30 (54.5)	131 (68.2)	2.34 (1.22–4.49)	**0.010**
No	36 (26.3)	25 (45.5)	61 (31.8)		
Prior treatment for STI, n = 158					
Yes	37 (31.6)	12 (29.30)	49 (31.0)	1.12 (0.51–2.43)	0.779
No	80 (68.4)	29 (70.70)	109 (69.0)		
Risky sexual behavior, n = 192§					
Yes	90 (65.7)	23 (41.8)	113 (58.9)	2.66 (1.40–5.06)	**0.002**
No	47 (34.3)	32 (58.2)	79 (41.1)		

*Boldface indicates statistical significance. OR, crude odds ratio; STI, sexually transmitted infection. †Variables are defined in the Appendix. ‡p values determined by χ^2^ tests. §Risky sexual behavior is defined as >1 of the following in the 3 mo before illness onset: condomless casual sex; multiple sexual partners (>2 more concurrent sex partners); treatment for sexually transmitted infections; transactional sex (payment for sex); sex with a sex worker.

### Epidemiologic Risk Factors among Children

All 71 children with known HIV status tested negative for HIV. Multivariate analysis indicated that the predictors of mpox positivity among children were contact with animals (aOR 9.97, 95% CI 1.27–78.34) and close contact with a person with a confirmed case (aOR 4.76, 95% CI 1.14–19.87) (Table 4). We did not include VZV in the model because of the high numbers of missing data. However, among the 64 children with known VZV status, the odds of being mpox positive were significantly higher among those who were VZV positive than among those who were VZV negative (OR 5.74, 95% CI 1.891–17.43).

**Table 4:** Epidemiologic risk factors for PCR-confirmed mpox among children (<18 y) during mpox outbreak, Nigeria 2022*

Variables		No. (%)			Univariate analysis			Multivariate analysis	
	Total	Mpox-pos	Mpox-neg		cOR (95% CI)	p value†		aOR (95% CI)	p value†
Sex at birth									
F	30 (41.1)	9 (34.6)	21 (44.7)		Referent			Referent	
M	43 (58.9)	17 (65.4)	26 (55.3)		1.53 (0.57, 4.11)	0.403		1.56 (0.46, 5.27)	0.477
Age group									
0–9 y	42 (57.5)	15 (57.7)	27 (57.4)		1.01 (0.38, 2.66)	0.984		2.03 (0.59, 7.05)	0.264
10–1 y	31 (42.5)	11 (42.3)	20 (42.6)		Referent			Referent	
Close contact confirmed									
Yes	13 (17.8)	7 (26.9)	6 (12.8)		2.52 (0.74, 8.51)	0.13‡		4.76 (1.14, 19.87)	**0.032**
No	60 (82.2)	19 (73.1)	41 (87.2)		Referent			Referent	
Animal exposure§									
Yes	7 (9.6)	5 (19.2)	2 (4.3)		5.36 (0.96, 29.91)	0.05‡		9.97 (1.27, 78.34)	**0.029**
No	66 (90.4)	21 (80.8)	45 (95.7)		Referent			Referent	
Place residence						0.632			
Rural	12 (16.4)	5 (19.2)	7 (14.9)		1.36 (0.35, 4.81)				
Urban/semi-urban	61 (83.6)	21 (80.8)	40 (85.1)		Referent				
VZV status¶						**0.001**			
Positive	24 (37.5)	15 (62.5)	9 (22.5)		5.74 (1.89, 17.43)				
Negative	40 (62.5)	9 (37.5)	31 (77.5)		Referent				

*Boldface indicates statistical significance. aOR, adjusted odds ratio; cOR, crude odds ratio; VZV, varicella zoster virus. †p values determined by χ^2^ tests except as indicated. ‡Fisher exact test. §The 5 mpox-pos children with animal exposures did not report associated contact with a human with suspected mpox. ¶Some VZV status data were missing.

## Discussion

Our study showed that laboratory-confirmed mpox was reported across various age groups and populations but was more common among persons who were young adult, male, and mostly urban or semi-urban dwellers. The demographic characteristics of the mpox-positive participants in our study are similar to those of the 2017–2019 mpox outbreak in Nigeria, which also predominantly affected young adult urban dwellers. Most cases of mpox during the 2022 outbreak in Europe and North America were among young adult urban dwellers, mostly GBMSM (*6**,**26*). In contrast, only 7.4% of the mpox participants in our study self-reported themselves as GBMSM; MPXV is probably not currently spreading within that particular social group in Nigeria. Another possibility is that cases of mpox in that group have either been overlooked or not accurately reported because GBMSM may avoid seeking clinical assessment because of laws in Nigeria that criminalize same-sex relationships.

The types of exposure settings reported in our study suggest human-to-human and zoonotic transmissions of MPXV during the 2022 outbreak in Nigeria. We identified independent epidemiologic risk factors for mpox positivity among study participants as having had close contact with a person with confirmed mpox and being in an adult age group. Specific risk factors for mpox among adults were >1 markers of risky sexual behaviors (e.g., multiple sex partners and condomless casual sex), and both sexual and nonsexual close contact with a person with suspected mpox. Among children, independent risk factors for mpox positivity were close contact with a person with confirmed mpox and contact with wild/domestic animals. Besides nonsexual physical contact, it might be postulated that mpox in Nigeria is also partly transmitted via risky sexual behavior among adults who subsequently transmit it to children through close contact. Various studies conducted outside Africa during the 2022 outbreak also identified risk factors for being mpox positive as having had multiple sex partners and other markers of risky sexual behavior (*9*,*11*,*12*,*14*). Similarly, since 2023, a cluster of clade I strain mpox cases in the DRC was linked to sexual contact, including among GBMSM (*21*).

Studies conducted mainly outside Africa suggest that ≈80% of mpox patients during the 2022 outbreak had sexual encounters before their diagnosis (*4*,*8*), and other studies conducted outside Africa have shown prior sexual activity to be associated with mpox infection among GBMSM and among heterosexual adults (*27*,*28*). The role of sexual contact and sexual behavior in the transmission of mpox was first proposed during the 2017–2019 mpox outbreak in Nigeria (*16*,*17*). A single-center study conducted during the 2022 outbreak in Nigeria reported mpox among linked heterosexual partners, suggesting a relationship between prior sexual contact and mpox infection in Nigeria (*29*). Our study, which was conducted on a national scale in Nigeria, corroborates the prior observations and supports a role of sexual activity in transmission of the MPXV among adults during the 2022 mpox outbreak in Nigeria.

With regard to animal exposure being independently associated with mpox positivity among children and not adults, it is plausible but not confirmatory that zoonotic transmission of MPXV in Nigeria is more common among children than adults. However, the large confidence interval of the OR related to animal exposure suggests uncertainty of that finding.

In our study, 18% of participants with available HIV test results had positive results, and odds of being mpox positive were 5 times higher among PLHIV than among those without HIV. During the 2022 global outbreak, 30%–50% of mpox-positive persons were PLHIV (*30*); various studies, including reports from Nigeria, have shown that those with advanced HIV have more severe disease and higher death rates than their HIV-negative counterparts (*31*). A review of 86 confirmed mpox cases during the 2017–2019 mpox outbreak showed that persons with mpox were ≈7 times more likely to be living with HIV than were those without mpox (*32*). Because of missing HIV test data, we cannot make definitive conclusions regarding HIV as an independent risk factor for mpox in Nigeria during the 2022 mpox outbreak. Even so, HIV and mpox are both sexually transmitted infections, which makes it plausible that risky sexual behavior might be a common factor for acquisition and further transmission of mpox.

Approximately half of the mpox-negative participants in our study were VZV positive, and ≈36% of mpox-positive participants also had a VZV-positive test result. We previously reported VZV co-infection to be independently associated with severe mpox during the 2022 mpox in Nigeria (*23*). The high prevalence of VZV co-infection among mpox-positive and mpox-negative participants reflects the endemicity of chickenpox, herpes zoster infection, or both in Nigeria and underscores that those VZV-related conditions are the main differential diagnoses for mpox in Nigeria. Of note, VZV co-infection was associated with higher odds of mpox among children but lower odds among adults. The reasons for the contrasting findings are not obvious from our study data. Because we did not distinguish chickenpox from reactivated herpes zoster virus infection in all participants, we could not classify the prevalence of those VZV-related conditions in relation to age, if any. Furthermore, we did not include VZV in the multivariate analysis because of a substantial amount of missing data, and as such, we could not confirm whether our findings were truly reflective of an age-related difference in the associations between VZV and mpox infections or if they resulted from the effects of another confounder. On the basis of the high rates of VZV-mpox co-infections observed from prior studies of mainly the clade I virus (*33*,*34*), it has been proposed but not confirmed that a breach in the skin caused by VZV lesions could increase the likelihood of transmission of MPXV and that MPXV may directly trigger VZV reactivation, resulting in herpes zoster virus infection (*34*,*35*).

The major limitations of our study are associated with recruitment of hospital-associated cases only, which could have led to underascertainment of mild mpox-positive cases and mpox-negative suspected cases in the community and missing data related to VZV and HIV co-infections among some participants, which precluded inclusion of these variables for multivariate analysis. The predominance of moderate to severe cases could also bias our study toward HIV-positive participants, given that they are more likely to have severe illness and thus need to seek care at or get admitted into healthcare facilities. We did not determine virus clades in our study, but prior epidemiologic data suggest that the 2022 mpox outbreak in Nigeria probably resulted from the MPXV clade IIb strain (*36*,*37*).

In conclusion, our study reveals that mpox primarily affects adults in Nigeria, often associated with sexual transmission, and that among children affected by mpox, the prominent drivers are animal contact and VZV infection. Our findings emphasize the value of addressing both sexual and nonsexual transmission routes in public health efforts to control the spread of mpox in Nigeria.

Appendix Additional information for study of mpox epidemiology and risk factors, Nigeria, 2022.
