# Supplementary material for: Mpox Epidemiology and Risk Factors, Nigeria, 2022
Source: Emerg Infect Dis. 2024 Sep;30(9):1799–808. doi: 10.3201/eid3009.240135 (PMC11346979; doi:10.3201/eid3009.240135)
Supplement: Appendix — Additional information for study of mpox epidemiology and risk factors, Nigeria, 2022. [file 24-0135-Techapp-s1.pdf]

Article DOI: https://doi.org/10.3201/eid3009.240135

EID cannot ensure accessibility for supplementary materials supplied by authors. Readers who have difficulty accessing supplementary content should contact the authors for assistance.

Epidemiology of and Risk Factors for Mpox, Nigeria, 2022

Appendix

Nigeria Centre for Disease Control Case Definition of Human Mpox

Suspected case: Any person presenting with a history of sudden onset of fever, followed by a vesiculopustular rash occurring mostly on the face, palms and soles of feet. Probable case: A probable case is defined as a case that met the clinical case definition and had an epidemiologic link to a confirmed case but was not laboratory confirmed. Confirmed case: A confirmed case is any case with laboratory confirmation of mpox by polymerase chain reaction (PCR) (*1*).

Laboratory Investigations

At least one type of skin specimen (either lesion swab or crust) was collected from each patient for laboratory confirmation of mpox and VZV infections by PCR. Tests for both infections were done centrally at the National Reference Laboratory of the Nigeria Centre for Disease Control Abuja, Nigeria as previously described (*2*). The detection of mpox and VZV is based on Taqman assay with primers and probes targeting mpox and VZV with the same thermal profile in one run. The two targets are detected in FAM channel, using Taqman buffer and U.S. CDC in-house primers and probe mastermix separately prepared for mpox, VZV and RNaseP in separate tubes. Other ancillary investigations were conducted depending on the patient clinical conditions.

Definitions of Study Variables

Animal exposure: defined as one or more of the following a month before illness onset; a: history of close contact with domestic animals (includes touching animal, animal bites or living in same household as animal); b: history of close contact with wildlife (includes touching, wildlife hunting, animal bite, and bush meat handling); c: history of consumption of bush meat.

Close contact with confirmed or suspected case: defined as one or more of the following in prior 1 month before illness onset: a: living in the same household as a confirmed/suspected case; b: sleeping on the same bed with confirmed/suspected case; c: physical contact with confirmed/suspected case.

Sexual contact with confirmed/suspected case: defined as physical contact with a confirmed or suspected case during sexual activity including oral, vaginal, and anal sex in the prior 1 month before illness onset.

Risky sexual behavior: defined as one or more of the following in the prior 3 months before illness onset: a: condomless causal sex in prior 3 months before illness; b: multiple sexual partners (two or more concurrent sexual partners); c: treatment for sexually transmitted infections; d: transactional sex (payment for sex); e: sex with a sex worker.

References

1. Nigeria Centre for Disease Control. Monkeypox outbreak response: interim national guidelines [cited 2024 Aug 2]. https://ncdc.gov.ng/themes/common/docs/protocols/50_1508912430.pdf

2. Ogoina D, Dalhat MM, Denue BA, Okowa M, Chika-Igwenyi NM, Yusuff HA, et al. Clinical characteristics and predictors of human mpox outcome during the 2022 outbreak in Nigeria: a cohort study. Lancet Infect Dis. 2023;23:1418–28.

Appendix Table. Age and occupational categories of study participants in relation to mpox-PCR status during mpox outbreak, Nigeria, 2022

Study variables	No. (%)		
	Mpox positive	Mpox negative	Total
Age group			
Adult (≥18 y)	137 (84.0)	55 (53.9)	192 (72.5)
Child (>1–17 y)	23 (14.1)	40 (39.2)	63 (23.8)
Infant (1–12 mo)	3 (1.8)	6 (5.9)	9 (3.4)
Neonate (<28 d)	0	1 (1.0)	1 (0.4)
Occupation*			
Artisan	9 (6.6)	2 (3.6)	11 (5.7)
Business/private sector	51 (37.2)	17 (30.9)	68 (35.4)
Government employed	35 (25.5)	13 (23.6)	48 (25.0)
Farmer/hunter	10 (7.3)	3 (5.5)	13 (6.8)
Healthcare worker	6 (4.4)	0	6 (3.1)
Student	19 (13.9)	17 (30.9)	36 (18.8)
Sex worker	3 (2.2)	0	3 (1.6)
Unemployed	4 (2.9)	3 (5.5)	7 (3.6)

*The occupation category includes adults only (n = 192).
